# Immunotherapy in Metastatic Mucosal Melanoma with Disseminated Intravascular Coagulation: A Case of Success

**DOI:** 10.1155/2021/5516004

**Published:** 2021-10-08

**Authors:** Helena Luna Pais, Paulo Luz, Soraia Lobo-Martins, André Mansinho, Rita Sousa, Rita Luís, Dolores Presa, Daniel Gomes, Luís Costa, Rita Teixeira de Sousa

**Affiliations:** ^1^Serviço de Oncologia, Hospital de Santa Maria, CHULN, Lisbon, Portugal; ^2^Serviço de Oncologia, Centro Hospitalar Universitário do Algarve, Faro, Portugal; ^3^CBIOS-Universidade Lusófona's Research Center for Biosciences & Health Technologies, Lisbon, Portugal; ^4^Serviço de Imagiologia, Hospital de Santa Maria, CHULN, Lisbon, Portugal; ^5^Serviço Anatomia Patológica, Hospital de Santa Maria, CHULN, Lisbon, Portugal; ^6^Serviço de Medicina Interna, Hospital de Santa Maria, CHULN, Lisbon, Portugal

## Abstract

Mucosal melanoma accounts for 1% of all melanomas. It is more aggressive than cutaneous melanoma, and local excision provides the best disease-free survival. The vast majority of patients eventually develop metastases, with a metastatic pattern independent of the primary tumor site. While studies show that BRAF and KIT inhibitors have a role in the management of these patients, the actual treatment focus is on immunotherapy. Herein is described the case of a 79-year-old woman with metastatic mucosal melanoma and bone marrow infiltration causing disseminated intravascular coagulation, who was treated with an immunotherapy combination (anti-CTLA-4 and anti-PD-1 antibodies), achieving complete disease remission. This is the third case of melanoma with disseminated intravascular coagulation at presentation and the second case treated with immunotherapy in the literature, but the only one achieving disease remission.

## 1. Introduction

Mucosal melanoma is a rare condition, accounting for 1% of all melanomas. It is usually located in the head and neck or anorectal or vulvovaginal regions (55%, 24%, and 18% of cases, respectively) but, sometimes, also in the urinary system, bladder, or small intestine. The average age at diagnosis is 70 years, although melanoma of the oral cavity frequently presents at younger ages [1]. These tumors have a worse prognosis than cutaneous melanoma. While the overall survival (OS) at 5 years is around 80% for cutaneous melanoma, it is only 25% for mucosal melanoma. To date, no risk factors for mucosal melanoma have been identified, nor a relationship with UVB ray exposure, as in cutaneous melanoma [1].

Regardless of the primary location, local excision offers the best disease-free survival opportunity for these patients. However, in most cases, tumor location (particularly in paranasal sinus tumors) or multifocal nature precludes complete resection with negative margins. Unfortunately, the vast majority of patients eventually develop metastases. A prospective analysis of 706 Asian patients with mucosal melanoma showed that the metastatic pattern is independent of the primary tumor site [2]. In the study, the most affected organs were regional lymph nodes (21.5%), followed by the lung (21%), liver (18.5%), and distant lymph nodes (9%), and 23% percent of patients were identified with stage IV at diagnosis.

These tumors also have biology different from that of cutaneous melanoma. BRAF mutations are identified in only 3–15% of cases compared to 50% in cutaneous melanoma, and c-KIT gene aberrations occur in 16–25% of cases compared to 5–10% in cutaneous melanoma. While some studies show that BRAF and KIT inhibitors have a role in the management of mucosal melanoma patients, the actual treatment focus is on immunotherapy [3, 4].

## 2. Case Report

Herein is reported the case of a 79-year-old female with a history of hypertension, hypothyroidism, and depression. She presented in October 2019 with a history of epistaxis in the previous four months, pain in the nasal, frontal, and parietal regions, and left eye ptosis. In one epistaxis episode, the woman went to the Emergency Department and was observed by an Otorhinolaryngology (ORL) specialist. Endoscopy showed multiple clots that were removed and a mass in the left nasal fossa aside the middle turbinate in the sphenoethmoidal recess. Incisional biopsies were performed, together with local hemostasis procedures.

**Figure 1:**
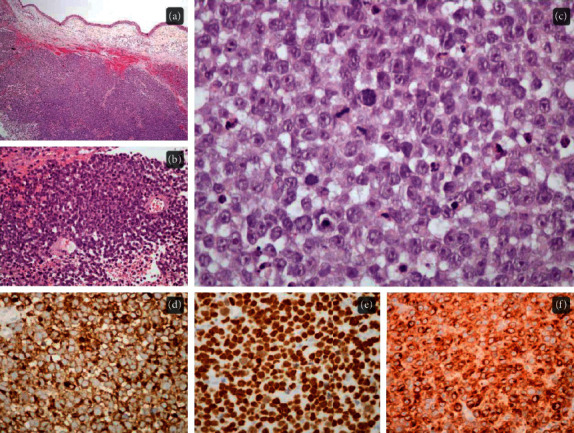
Microscopically, a solid tumor infiltrates the submucosa ((a); H&E, 40x), displaying areas of geographic necrosis ((b); H&E, 200x). On higher magnification ((c); H&E, 400x), cherry-red nucleoli are conspicuous and numerous mitosis and apoptosis figures can be recognized. Neoplastic cells were positive for HMB-45 ((d); 400x), SOX-10 ((e); 400x), and Melan-A ((f); 400x) immunostaining.

Histological examination revealed extensive submucosal infiltration by a sheet of round cells with enlarged nuclei, prominent nucleoli, numerous mitosis, and geographic necrosis. Ancillary studies were conducted, showing immunoreactivity for Melan-A, SOX-10, HMB-45, MiTF, and nestin. In addition, cells were negative for cytokeratin, chromogranin A, synaptophysin, NSE, CD3, CD20, CD5, CD56, vimentin, desmin, myogenin, CD99, and EBER during in situ hybridization. Histomorphology and immunohistochemical profiles suggested the diagnosis of mucosal melanoma (Figure 1).

**Figure 2:**
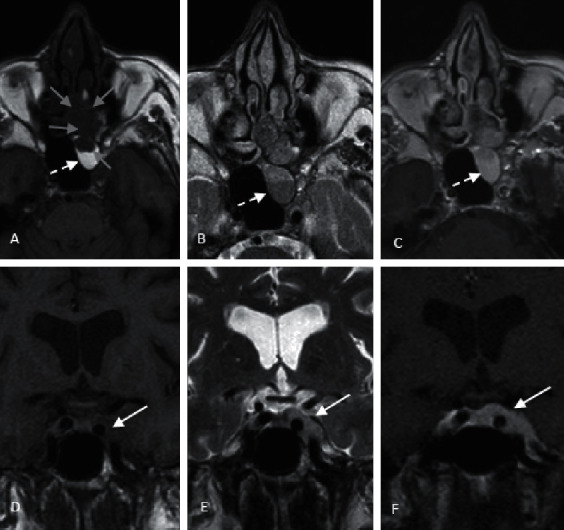
Initial MRI images: (a, c) axial and coronal T1WI, (b, e) axial and coronal T2WI, and (c, f) axial and coronal T1WI after gadolinium intravenous administration. The left nasal, ethmoidal, and sphenoidal lesion (grey arrows) can be seen with cavernous extension (solid white arrows), with low signal on T1WI and T2WI and homogeneous enhancement after contrast administration, with sinus obstruction and retention of secretions in ethmoid and sphenoid sinuses (dashed white arrows). MRI, magnetic resonance imaging; WI, weighted image.

Computed tomography (CT) and magnetic resonance imaging (MRI) identified a 4 cm × 2.2 cm × 3.7 cm lesion in the posterior half of the left nasal fossa (Figure 2). The lesion presented locoregional extension internally to the nasal fossa, shifting the nasal septa to the right and laterally, focal widening of ethmoidal cells with lamina papyracea disruption, and extension to the orbital apex and sphenoid slit. Posteriorly, it extended to the left cavernous sinus.

The case was discussed in the ORL multidisciplinary meeting, and a systemic therapy was proposed due to lesion irresectability.

At presentation, the patient had ECOG performance status 2 and left ptosis, with no other relevant alterations on physical examination. Thoracoabdominopelvic (TAP) CT scan showed peritoneal implants, and BRAF and c-KIT mutation screening disclosed no mutations.

Due to continuous epistaxis episodes, the patient was proposed for hemostatic radiotherapy (RT). She underwent 5 RT sessions with 20 Gy each. After hemostatic RT, she experienced no additional epistaxis episodes but maintained progressive red blood cell count decline and was referred to immunohemotherapy consultation for transfusional support and darbepoetin therapy. Pain improvement enabled analgesia de-escalation and lactate dehydrogenase (LDH) reduction from 3506 U/L to 2230 U/L.

Systemic treatment was proposed with off-label nivolumab 3 mg/kg plus ipilimumab 1 mg/kg every 3 weeks for four doses, as per the Checkmate 511 study protocol. Although there is currently no robust evidence of the efficacy of this regimen in mucosal melanoma, a phase II study suggested that it is less toxic and has no meaningful efficacy differences compared to cutaneous melanoma [5]. The patient signed informed consent, and treatment was started in December 2019. At the start of treatment with anti-PD-1 plus anti-CTLA4, the serum LDH level was 2172 U/L (normal range, 100−250 U/L), fibrinogen level was 157 mg/dL (normal range, 200−400 mg/dL), hemoglobin (Hg) level was 8 g/dL, and platelet (plat) count was 83 × 10^9^/L.

After the first immunotherapy cycle, the patient reported pain reduction and ptosis improvement. Blood work showed a decrease in the LDH level to 503 U/L, as well as anemia (Hg 6.4 g/dL), leucopenia (leuc 2.88 × 10^9^/L), thrombocytopenia (plat 35 × 10^9^/L), fibrinogen consumption (78 mg/dL), and D-dimer (13.83 ug/mL) (normal range, 0.0–0.5 ug/mL). No major alterations in prothrombin time and activated partial thromboplastin time were observed (14 and 29 seconds, respectively), as well as there were no signs of hemorrhagic dyscrasia. The patient was admitted to the hospital with the diagnosis of disseminated intravascular coagulation (DIC). Bone marrow aspirate was performed, showing hypocellular marrow with hypoplasia of the three hematopoietic lineages and infiltration by nonhematopoietic atypic cells, compatible with metastases. Support therapy with erythrocyte concentrate and fibrinogen was provided, achieving clinical stabilization. The patient maintained treatment with nivolumab and ipilimumab, completing four cycles in February 2020, with complete ptosis and headache remission. LDH progressively decreased, reaching 244 U/L in March 2020. Since then, she is under maintenance therapy with nivolumab 480 mg every 4 weeks. Reported adverse events included vitiligo in June 2020 and hypothyroidism (TSH 12.7 *µ*U/ml and FT4 0.78 ng/dL; normal range 0.30–4.20 *µ*U/mL and 0.85–1.70 ng/dL, respectively), with the need for thyroid medication adjustment. The last TAP CT scan, performed in June 2020, showed complete peritoneal carcinomatosis remission, and cranial MRI performed in August 2020 also showed no signs of disease, confirming complete disease remission according to RECIST 1.1 criteria.

## 3. Discussion

This report describes the second case of DIC in a patient with metastatic melanoma treated with immunotherapy, but the first case affecting the mucosa. Gbadamosi et al. [6] reported a case of bone marrow metastatic melanoma presenting with DIC. The patient had an excellent response to combined immune-checkpoint inhibitors nivolumab and ipilimumab.

All DIC cases reported so far were in patients with metastatic disease and associated with dismal prognosis (OS ranging from weeks to approximately 18 months). DIC may present in acute and chronic form, with thrombotic or bleeding complications and with asymptomatic laboratory abnormalities [7]. The pathogenesis of cancer-related DIC is complex and multifactorial, being characterized by activation of the blood coagulation system, excessive consumption of hemostatic factors, and secondary fibrinolysis. DIC is a rare melanoma complication resulting from the increased expression of coagulation and fibrinolysis markers—like factor V, factor VII, von Willebrand factor, plasminogen, and D-dimer—and low platelet counts [8]. In a systematic review about DIC and melanoma, most patients presented with bleeding [9].

DIC management comprises three main approaches: supportive therapy with blood products, pharmacological treatment targeting coagulation or fibrinolytic abnormalities, and, most importantly, rapid and aggressive treatment of the underlying condition [7]. Data about the best approach for patients with mucosal melanoma and this complication and how it can affect their prognosis are currently lacking [9].

In 2017, D'Angelo et al. [10] published a pooled analysis of six clinical trials of patients diagnosed with advanced mucosal melanoma treated with immunotherapy (nivolumab alone or combined with ipilimumab). Eighty-six patients treated with nivolumab monotherapy and 35 patients treated with nivolumab and ipilimumab were included. The analysis conﬁrmed that patients with metastatic mucosal melanoma can beneﬁt from immunotherapy, albeit with lower objective response rates (ORRs) than reported in cutaneous melanoma. With nivolumab monotherapy, ORR was 23.3% (95% CI, 14.8–33.6%) for mucosal melanoma and 40.9% (95% CI, 37.1–44.7%) for cutaneous melanoma and progression-free survival (PFS) was 3.0 months (95% CI, 2.2–5.4 months) and 6.2 months (95% CI, 5.1–7.5 months), respectively. With the combination of nivolumab (1 mg/kg) and ipilimumab (3 mg/kg), ORR was only 37.1% (95% CI, 21.5–55.5%) in patients with mucosal disease compared with 60.4% in patients with cutaneous disease (95% CI, 54.9–65.8%) and PFS was 5.9 months (95% CI, 2.8 months−not reached) and 11.7 months (95% CI, 8.9–16.7 months), respectively. Patients in these trials were treated with a schedule different from that of the current patient, who received nivolumab 3 mg/kg and ipilimumab 1 mg/kg. Although no data are currently available about this regimen in mucosal melanoma, it showed to be less toxic and have no meaningful efficacy differences compared to the observed ones in cutaneous melanoma, potentially representing a useful option for frail patients [10]. This is also the first report of metastatic mucosal melanoma treated with the combination of nivolumab 3 mg/kg and ipilimumab 1 mg/kg.

In an analysis of 84 patients with metastatic mucosal melanoma (51 of whom were ipilimumab-naïve) treated with pembrolizumab in three clinical trials (KEYNOTE-001, -002, -006), ORR was 19% (95% CI, 11−29%), with an average response time of 27.6 months, PFS was 2.8 months (95% CI, 2.7–2.8), and OS was 11.3 months (95% CI, 7.7–16.6) [11].

The response rates to immunotherapy in mucosal melanoma are diminished, which can be related to lower PD-L1 expression in these tumors. In the analysis by D'Angelo et al. [10], the proportion of mucosal melanomas with PD-L1 expression greater than 5% was 21% compared to 35% in cutaneous melanoma. Another explanation may be related to tumor mutational burden. In general, the number of tumor mutations is related to neoantigen formation and hence to an increased response to immunotherapy. Mucosal melanoma is a tumor with a low mutational burden compared to cutaneous melanoma, probably for not being induced by sun exposure [3].

Results of the only clinical trial specifically designed for metastatic mucosal melanoma were published in February 2021. A total of 20 patients were included in this phase II trial and treated with nivolumab (2 mg/kg every 3 weeks). The primary endpoint, ORR, was 23.5% (one complete response, three partial responses, and five stable diseases), PFS was 1.4 months (95% CI, 1.2–2.8), and OS was 12 months (95% CI, 3.5−not reached) [12].

In summary, as far as the authors are aware, this is the first case of metastatic mucosal melanoma presenting with DIC achieving complete remission with immunotherapy, in the literature. DIC is a rare and serious melanoma complication, requiring rapid response for a favorable outcome. Despite the lack of robust data in the literature, the present case suggests that the combination of nivolumab and ipilimumab is a treatment option, not only for cutaneous melanoma but also for mucosal melanoma. A better understanding of the biology of mucosal melanoma may help to select patients who benefit the most from immunotherapy.

## Data Availability

The primary data about the patients were obtained from the electronic medical record of Centro Hospitalar Universitário Lisboa Norte. Cited manuscripts were found on PubMed.
